# Postpartum evolution of newborns to mothers addicted to “new drugs” and recently diagnosed with HIV infection

**DOI:** 10.1186/1471-2334-13-S1-O2

**Published:** 2013-12-16

**Authors:** Rodica Ungurianu, Mariana Mărdărescu, Adrian Streinu-Cercel, Ioana Anca, Cristina Petre, Ruxandra Drăghicenoiu, Adrian Abagiu, Sorin Petrea, Carina Matei, Cristian Anghelina, Alina Cibea, Ileana Leu, Mihai Mitran, Dan Oțelea, Alexandra Mărdărescu

**Affiliations:** 1 National Institute for Infectious Diseases “Prof. Dr. Matei Balş”, Bucharest, Romania; 2 Carol Davila University of Medicine and Pharmacy, Bucharest, Romania; 3 The Institute for Mother and Child Protection “Alfred Rusescu”, Bucharest, Romania; 4 Clinical Hospital for Obstetrics and Gynecology “Prof. Dr. Panait Sârbu”, Bucharest, Romania; 5 Romanian HIV/AIDS Centre, National Institute for Infectious Diseases “Prof. Dr. Matei Balş”, Bucharest, Romania

## Background

In 2011 and 2012 Romania registered a significant increase in the number of intravenous drug users (IVDUs) along with a change in the use pattern, namely the replacement of heroin with the so called “new drugs”. These are mostly synthetic cannabinoids and cathiones. Within this context, the share of new IVDU-HIV cases expanded from 3% in 2010 to 29% in 2012. Our objective was to observe the evolution of 30 newborns to mothers recently diagnosed with HIV infection, who were also “new drugs” consumers.

## Methods

During 1 January 2011 – 31 December 2012 we assessed 30 newborns exposed to HIV and “new drugs” by clinical and biological screenings. Relevant data were recorded: gender, age, moment of diagnosis, antiretroviral treatment (ART) prophylaxis, type of birth, type of nourishment, CD4 count, viral load (VL), and ultrasound evaluation. For mothers we focused on age, time of HIV diagnosis, treatment/prophylaxis, type of birth, type of consumed drugs.

## Results

The number of newborns exposed perinatally to HIV/HBV/HCV/drugs and new drugs increased in 2011-2012 compared to previous years. They presented several neo-natal problems of adjustment especially a severe withdrawal syndrome from the very first hours of life with life threatening risks. From the evaluated children, 57% presented withdrawal syndrome, 53% neurological lesions, 27% various cardiac malformations, and 2% rare diseases.

## Conclusion

Most of these pregnant women, who are HIV positive and drug consumers, especially of “new drugs” do not access routine check-ups, this explaining their absence from records as drug users; also, if they are diagnosed with HIV during pregnancy they refuse to take specific treatment. Due to the shift in drug use, the effect on newborns perinatally exposed to HIV cannot yet be fully grasped and represents a key concern for professionals working with this pathology. From a social perspective, these women are either very poor or come from dysfunctional families, who are reluctant in providing them with the proper support.

